# Aggressive juvenile fibromatosis of the paranasal sinuses: case report and brief review

**DOI:** 10.1186/1756-8722-1-3

**Published:** 2008-05-28

**Authors:** Shaheen E Lakhan, Robert M Eager, Lindsey Harle

**Affiliations:** 1 Executive Director, Global Neuroscience Initiative Foundation, Los Angeles, CA, USA; 2 Research Consultant, Department of Biomedical Sciences, Global Neuroscience Initiative Foundation, Los Angeles, CA, USA

## Abstract

Desmoid fibromatoses are benign, slow growing fibroblastic neoplasms, arising from musculoaponeurotic stromal elements. Desmoids are characterized by local invasion, with a high rate of local recurrence and a tendency to destroy adjacent structures and organs. Desmoid fibromatoses are rare in children, and though they may occur in the head and neck region, are extremely rare in the paranasal sinuses. Here we report a case of extraabdominal desmoid fibromatosis in a seven-year-old boy involving the sphenoid sinus, one of only six published reports of desmoid fibromatosis of the paranasal sinuses. The expansile soft tissue mass eroded the walls of the sphenoid sinus as well as the posterior ethmoid air cells extending cephalad through the base of the skull. We discuss the clinicopathologic features of this lesion, including structural and ultrastructural characteristics, and we review the literature regarding treatment and outcome.

## Background

Desmoid tumors arise from musculoaponeurotic stromal elements and are locally invasive, deep-seated fibrotic tumors. They are destructive of surrounding tissue, with a high rate of recurrence, but are not known to have the capacity to metastasize. Desmoid tumors have two general classifications, intraabdominal and extraabdominal. This distinction is significant in determining proper clinical management. Extraabdominal tumors are predominantly sporadic, and often can be effectively treated with local resection; systemic treatment is generally reserved for refractory tumors. Conversely, intraabdominal desmoid fibromatosis, for example those seen with familial adenomatous polyposis and Gardner syndrome, are often diffusely infiltrative and surgically unresectable; systemic therapy is considered first-line treatment of intraabdominal desmoids. Extraabdominal tumors in the paranasal sinus are extremely rare; to the best of our knowledge only six cases have been reported in the literature [1-6] (Table 1).

**Table 1:** Reported cases of pediatric desmoid fibromatosis of the paranasal sinuses.

**Age**	**Location**	**Presentation**	**Pathology**	**Therapy**	**Response**	**Reference**
2 year old male	Right maxillary sinus	Nasal obstruction	Aggressive fibromatosis	Surgical resection	Lost to follow up	[1]
14 year old female	Right parotid/mandible	Right facial deformity	Aggressive fibromatosis	Surgical resection (positive margins)	No recurrence at < 1 year	[6]
15 month old male	Nasal cavity/anterior maxilla	Facial deformity	Aggressive fibromatosis	1. Surgical resection (positive margins) 2. Surgical resection (negative margins)	Recurrence in 1 month, no recurrence	[5]
2 year old male	Left maxillary sinus	Nasal deformity	Desmoid fibromatosis	Surgical resection (twice), followed by adjuvant tamoxifen	No recurrence at 2 years	[4]

Conley et al. [2] reported a series of 40 different cases, three cases between the ages 1–10. One of these cases involved the ethmoid sinus.

Fu [3] reported two cases of juvenile fibromatosis ages 2 and 10. One of these cases involved the maxillary sinus.

## Case presentation

An otherwise healthy seven-year-old male presented with a six month history of chronic sinus congestion and halitosis. He was initially treated for atopy and bacterial sinusitis with no resolution of symptoms. Suspicion was raised of a foreign body in the nose and ENT consultation was ordered. Prior to endoscopic removal of the foreign body, computed tomography (CT) of the head was performed.

CT revealed a large expansile mass, 4 cm in greatest dimension, expanding and eroding the walls of the sphenoid sinus and the posterior ethmoid air cells. Because the mass extended cephalad into the base of the skull, magnetic resonance imaging (MRI) was performed. MRI found no evidence of meningeal involvement or brain parenchymal invasion and the major intracranial arteries appeared intact. The right optic nerve was displaced but without evidence of impingement.

**Figure 1:**
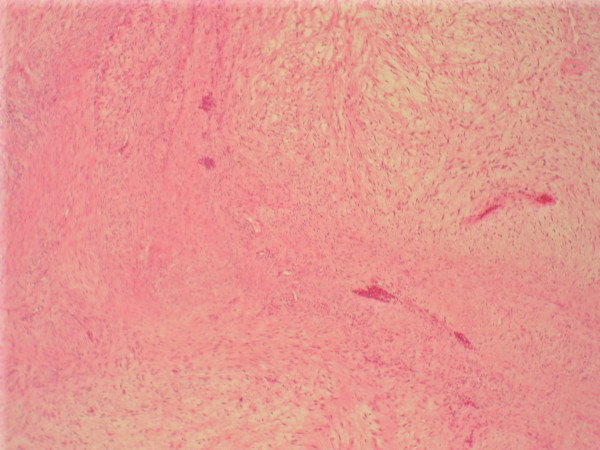
**Paranasal tumor, H&E stained section, low power (10×)**. The section shows a spindle cell neoplasm with tapering nuclei, eosinophilic cytoplasm and minimal atypia. Focally, myxoid features predominated.

**Figure 2:**
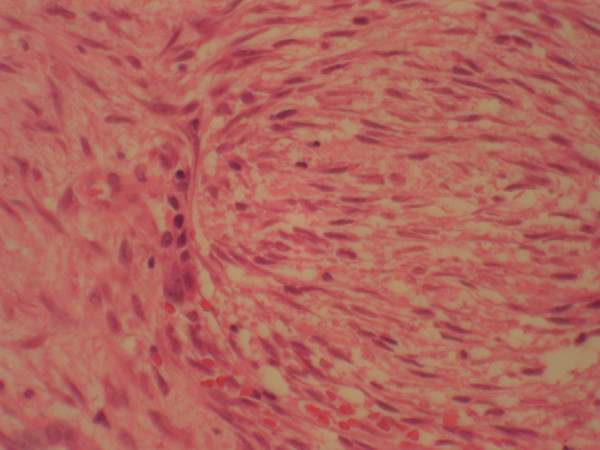
Paranasal tumor, H&E stained section, high power (40×).

The patient underwent functional endoscopic sinus surgery with biopsy of the lesion. Histological analysis revealed a cellular myofibroblastic neoplasm suggestive of extraabdominal desmoid fibromatosis (Figures 1, 2). Surgical resection was performed and histological analysis confirmed the diagnosis. Surgical margins were positive. Because of the rarity of this tumor, particularly in the paranasal sinuses of a child, immunohistochemical examination was performed. The tumor showed focal positivity for SMA and multifocal nuclear positivity for beta catenin; desmin, S-100, and CD34 were negative.

## Discussion

Desmoid tumors are rare, accounting for approximately 0.03% of all neoplasms, and less than 3% of all soft tissue tumors. The estimated incidence in the general population is 2-4/1,000,000/year, which in the US translates to approximately 900 new tumors annually [7]. Individuals between the ages of 15 and 60 are most often affected; desmoid tumors are rare in the young and in the elderly. They are slightly more common in women than in men [8,9], and there is no significant racial or ethnic distribution. Desmoids tend to be large bulky tumors that locally infiltrate adjacent tissue structures. Histologically, they are characterized by small bundles of spindle cells in an abundant fibrous stroma. The fibroblasts have a propensity to concentrate at the periphery of the lesion, and the cellularity is low. There are usually few mitotic figures and necrosis is absent. The etiology of desmoid tumors is unknown. However, the identification of clonal chromosomal changes in a significant fraction of cases supports the neoplastic nature of these tumors [10], and emerging evidence implicates dysregulated wound healing in the pathogenesis of these and other fibroblastic lesions. Trisomy 8 and 20 as nonrandom clonal chromosomal changes, particularly trisomy 8, occur in at least 30% of sporadic desmoid tumors [11-14]. Although the clinical relevance of these genetic abnormalities is unclear, these genetic insults appear to be associated with a higher risk of recurrence [12].

## Treatment

Because of their locally infiltrative nature, desmoid tumors are traditionally treated by local resection with wide surgical margins when significant morbidity can be avoided [15,16]. Considering the potential toxicity and morbidity associated with local and systemic therapy in children, complete surgical excision is the treatment of choice for aggressive juvenile fibromatosis. Because these are benign tumors with a high rate of recurrence, surgeons must balance the need to obtain tumor-free margins while at the same time using function-preserving approaches to minimize major functional and cosmetic sequelae. The available data are conflicting with regard to the importance of complete resection. Buitendijk et al. [17] reported that, of 187 published cases of juvenile fibromatosis, the single greatest determinant of tumor recurrence was incomplete resection. In another evaluation of 63 pediatric patients, the only factor associated with an increased rate of recurrence-free survival was negative surgical margins (70% versus 15% with positive margins) [18]. In contrast, several authors report that the risk of recurrence is independent of margin status [19-25]. In one of the largest series of 203 patients undergoing surgery for either primary or recurrent desmoid tumors, margins were microscopically positive in 57 and negative in 146 [21]. As expected, the disease-free survival rate was significantly better in patients with primary disease (76% versus 59% at 10 years), but it was not significantly worse for those with microscopically positive versus negative margins at primary surgery (five year disease-free survival rate for those with positive and negative margins, 79% versus 82%; at 10 years, 74% versus 77%). In patients who undergo aggressive resection with wide margins recurrence rates remain at 23% to 39% [15,26-29]. When they recur, salvage therapy with radiation therapy (RT) and/or repeat excision is often successful. This data cast some doubt on the current dogma of aggressive pursuit of negative surgical margins in cases that may result in excessive morbidity [9,19]. The uncertainty as to the importance of positive resection margins also spurs controversy with regard to the role of postoperative RT for patients with incompletely resected disease.

### Radiation therapy

In patients for whom surgery is not an option, primary RT is an effective alternative therapeutic course. In several reports, RT alone (50 to 60 Gy) or combined with surgery in patients with positive resection margins achieves long-term control in approximately 70% to 80% of patients with desmoids [23,25,27,29-31]. The volume of disease does not appear to affect the probability of local control. Local recurrence rates do not appear to correlate with the use of higher doses. In one study of 23 patients the relapse rate at five years was 31%, and radiation doses above 56 Gy did not improve outcome. In fact, higher dose levels were associated with more complications: 30% (high dose) versus 5% (low doses) at 15 years [23]. Adverse events included the following: soft tissue necrosis, bone fracture, radiation enteritis, peripheral neuropathy, edema with cellulitis, limb shortening, and bone hypoplasia. Positive resection margins were not a prognostic factor in this report.

### Systemic Therapy

Patients with extraabdominal desmoids and multiple locoregional recurrences despite adequate surgical and/or radiation treatment are generally considered for systemic therapy. Other indications for systemic therapy include unresectable tumors and intraabdominal desmoids. In these settings, early and aggressive systemic therapy is important to avoid life-threatening complications. A variety of agents are active, including noncytotoxic therapy (i.e. non-steroidal anti-inflammatory drugs (NSAIDs), hormone manipulation, and pirfenidone) and cytotoxic chemotherapy. The conclusions that can be drawn as to the relative effectiveness of these agents in the treatment of desmoid tumors are limited by the low incidence. Unfortunately the majority of data generated on this topic consists of case reports. Therefore, in the absence of clear evidence, a conservative approach is appropriate. In cases where there is no impending threat to life or function it is reasonable to begin with less toxic approaches, such as hormone therapy or NSAIDs. Cytotoxic chemotherapy is a more appropriate choice for patients with rapidly growing tumors or those who are highly symptomatic.

### Noncytotoxic systemic therapy

Clinical and experimental evidence suggest the hormone dependency of desmoid growth. Clinical benefit is reported in nearly 50% of patients with tamoxifen treatment, with most of the objective responses being partial rather than complete. Tumors are slow to manifest an actual reduction in size, and not infrequently, shrinkage lags behind discontinuation of therapy by months or even years. Response durations vary to a great degree, ranging from seven months to 12 years [32]. The mechanism is unclear since response to treatment does not appear to correlate with the presence of estrogen receptor alpha [9,33], and the significant lag of the therapeutic response has led some to hypothesis that the mechanism of action is estrogen independent [46]. There are also documented responses to NSAIDs, most often sulindac, both alone and in combination with tamoxifen [9,34-39]. At least one report documents the resolution of a desmoid tumor being treated with indomethacin and ascorbic acid for 14 months [40]. Although response rates as high as 70% are reported with combined therapy [9], regression is usually partial and may take many months after an initial period of tumor enlargement. In addition, response criteria for these case reports are not standardized. Several case reports describe objective response or prolonged periods of disease stabilization with interferon alpha (IFN-alpha) [41-43], in some cases following failure of sulindac and tamoxifen [36,44,45]. However, new data suggesting that IFN type I signaling is a positive regulator of neoplastic growth has raised questions about the therapeutic role of IFN-alpha in this disease [46]. An increasing number of reports suggest clinical and radiographic benefit from the tyrosine kinase inhibitor imatinib (Gleevec) [47,48]. This effect is presumably due to tumor expression of activated receptor tyrosine kinases c-kit and/or platelet-derived growth factor receptor-alpha (PDGFRA). However the clinical efficacy of imatinib and the mechanism underlying clinical benefit in the patients who have been treated with this agent are uncertain.

### Cytotoxic systemic therapy

Although desmoid tumors as a group are generally slow growing with low metastatic potential, there are several highly active chemotherapy regimens that can potentially produce durable response. The combination of low dose methotrexate and vinblastine has shown promising results, particularly in children [49-52]. One study of 30 patients with a median age of 27 reported 10 year progression free survival in 67% [49]. Liposomal doxorubicin has proven to be a well tolerated and efficacious option [53]. High dose doxorubicin or ifosfamide-based regimens have shown more activity and increased incidence of serious toxicity; thus they are usually reserved for cases that are life threatening and refractory to other treatments [54-57].

## Conclusion

Desmoid fibromatosis are rare pediatric tumors, and the case reported here is one of only six published accounts of pediatric desmoid fibromatosis of the paranasal sinuses. Aggressive juvenile fibromatoses are a group of lesions with variable response to treatment; they are locally aggressive but have low metastatic potential. Current treatment ranges from traditional surgical resection to multidisciplinary approaches involving local radiation and/or systemic cytotoxic and cytostatic agents. However, surgical resection with wide margins remains the primary treatment for extraabdominal fibromatoses. Reports in the literature are conflicting as to the importance of obtaining tumor-free surgical margins; some retrospective analyses have found a significant decrease in recurrence rate with negative margins, while others have not. Based on these reports, the optimal treatment strategy for pediatric desmoids fibromatoses is patient-dependent, and clinical decisions must be made based upon tumor location, risk of surgical morbidity and risk of radiation-induced damage. Radiation therapy and cytotoxic chemotherapy in pediatric patients should be used in cases that are refractory to surgery and noncytotoxic systemic therapy due to the potential of growth disturbance, contracture, and the development of secondary malignancy.

## Abbreviations

CT: computed tomography; IFN-alpha: interferon alpha; MRI: magnetic resonance imaging; NSAID: non-steroidal anti-inflammatory drug; PDGFRA: platelet-derived growth factor receptor-alpha; RT: radiation therapy; SMA: smooth-muscle actin.

## Competing interests

The authors declare that they have no competing interests.

## Authors' contributions

SL, RE, and LH secured the case, conducted the literature review, and participated in the preparation of the manuscript. All authors read and approved the final manuscript.

## Consent

Written informed consent was obtained from the patient's parents for publication of this case report and any accompanying images. A copy of the written consent is available for review by the Editor-in-Chief of this journal.
